# Dose-effect relationship of linear accelerator based stereotactic radiotherapy for brain metastases

**DOI:** 10.1186/s13014-023-02360-y

**Published:** 2023-10-30

**Authors:** Ning Wu, Zhiqiang Wang, Xin Guo, Hongfu Zhao

**Affiliations:** Department of Radiation Oncology, China-Japan Union Hospital of Jilin University, No. 126, Xiantai Street, 130033 Changchun City, Jilin PR China

**Keywords:** Brain metastases, Linear accelerator, Stereotactic radiotherapy, Dose-effect relationship

## Abstract

**Objective:**

The purpose of this study is to reveal the dose-effect relationship of linear accelerator (LINAC)-based stereotactic radiotherapy (SRT) in patients with brain metastases (BM).

**Materials and methods:**

The PubMed, Cochrane, and Web of Science databases were used to identify studies that reported local tumour control after LINAC-based SRT in patients with BMs. Studies of other approaches that could affect local tumour control, such as whole brain radiotherapy, targeted therapy, and immunotherapy, were excluded from the dose-effect relationship analysis. Data extracted included patient and treatment characteristics and tumour local control. Probit model in XLSTAT 2016 was used for regression analysis, and *P* < 0.05 was set as the statistically significant level.

**Results:**

After literature screening, 19 eligible studies involving 1523 patients were included in the probit model regression analysis. There was no significant dose-effect relationship between nominal BED_10_ and peripheral BED_10_ versus 12-month local control probability. There were significant dose effect relationships between the centre BED_10_ and the average BED_10_ versus the 12-month local control probability, with *P* values of 0.015 and 0.011, respectively. According to the model, the central BED_10_ and the average BED_10_ corresponding to probabilities of 90% 12-month local control were 109.2 Gy_BED10_ (95% confidence interval (CI): 88.7–245.9 Gy_BED10_) and 87.8 Gy_BED10_ (95% CI: 74.3–161.5 Gy_BED10_), respectively. A 12-month local control rate of 86.9% (95% CI: 81.7–89.7%) and 85.5% (95% CI: 81.2–89.2%) can be expected at a centre BED_10_ of 80 Gy and an average BED_10_ of 60 Gy, respectively.

**Conclusion:**

For patients with BM treated with LINAC-based SRT, more attention should be given to the central and average doses of PTV. A clear definition of the dose prescription should be established to ensure the effectiveness and comparability of treatment.

**Supplementary Information:**

The online version contains supplementary material available at 10.1186/s13014-023-02360-y.

## Introduction

Brain metastases (BM) occur in up to one-third of adult patients with solid tumour malignancies and lead to considerable patient morbidity, anxiety, and mortality [1]. The rising incidence of brain metastases has been ascribed to the development of better imaging and screening techniques and the formulation of better systemic therapies. Radiotherapy is the most common form of local therapy used in patients with BM. Whole-brain radiotherapy (WBRT) has long been considered the cornerstone of treatment to relieve symptoms and control tumours. However, WBRT is associated with cognitive dysfunction, balance problems, and hearing loss, as well as several acute and late side effects, such as fatigue, alopecia, anorexia, xerostomia, and nausea [2].

Several comparative studies showed that stereotactic radiotherapy (SRT) showed advantages in tumour control or quality of life for patients with BM compared with WBRT [3–5]. In SRT for patients with BM can be delivered as either a single fraction (usually 18 to 24 Gy) of highly conformal and high-dose treatment or as a fractionated SRT, ranging from 24 to 30 Gy in 3 fractions or 25 to 35 Gy in 5 fractions [6]. Both the physical dose and biological equivalence dose (BED) of the prescription dose vary greatly. Several studies have attempted to determine the dose-effect relationship between physical dose or BED and local control in SRT for patients with BM and have obtained valuable information [6–9]. With the implementation and progress of LINAC-based SRT for patients with BM, studies including dose parameters in detail and tumour control have been increasingly reported. The purpose of this study was to reveal the dose-effect relationship of LINAC-based SRT for patients with BM.

## Materials and methods

### Data sources and search strategy

A comprehensive literature search was conducted using the PubMed, Web of Science and Cochrane databases to determine the published articles regarding BM patients treated with SRT. The title field were searched for “stereotactic radiosurgery,“ “stereotactic radiotherapy,“ “hypofractionated radiosurgery,“ “stereotactic body radiotherapy,“ “stereotactic ablative radiotherapy,“ “SRT,“ “SRS,“ “SBRT,“ and “SABR” to determine article set A on SRT. Similarly, the title field was searched for “brain metastases” and “brain metastasis” to determine article set B about BM. The intersection of A and B was used to determine the articles about patients with BM treated with SRT. Our last literature search was on Mar 17, 2023 (see Additional File 1: Table S1).

### Inclusion criteria

1. The treatment conforms to the SRT technical specifications in the AAPM Task Group 101 report. For example, the dose per fraction is 6 to 30 Gy, and the number of fractions is 1 to 5.
2. Original studies that reported the prescription dose and 6-month and/or 12-month local tumour control rate, were included.

### Exclusion criteria

Stereotactic radiotherapy is not implemented based on C-arm-mounted LINAC but is based on CyberKnife radiosurgery system, Gamma Knife, etc.
Combined with other treatment methods that may change the outcome of tumour local control, such as whole brain radiotherapy, surgery, targeted therapy, or immunotherapy, etc.
3. Studies about SRT as salvage treatment in patients with recurrent BM.

### Data extraction

After removing the duplicates, we screened the literature by title and abstract, and the remaining literature was screened by full text. When the patient data reported in the literature overlapped, we selected the latest and most complete data. The bibliographic references of relevant reviews have also been reviewed and included according to the criteria. When the data originated from overlapping or almost the same patients, the most recent and comprehensive articles were included. Literature screening and data extraction were conducted independently by two authors according to the inclusion and exclusion criteria, and objections were resolved through negotiation.

The following data from all enrolled studies were extracted: (1) Study information: first author, publication year, country, and inclusion time of patients; (2) Patients and tumour characteristics: number of patients, number of metastases, median or mean age, most common histology, tumour size, and extracranial disease control; (3) Treatment characteristics: the margin from gross target volume (GTV) to planning target volume (PTV), prescription dose, peripheral dose, central dose, and median number of fractions; and treatment technology; (4) Clinical outcomes: median follow-up and local control (LC) at 6 months and 12 months. The biologically effective doses (BED) were calculated using the linear quadratic (LQ) equation: BED_10_ = n×d×[1 + d/(α/β)], where d represents the fraction dose, n represents the fractions. The α/β value quantifies the sensitivity to changes in fraction size, and higher α/β values (7 to 20 Gy) are typical values for tumor control, showing lower effects of fractionation. Referring to the dose-response effect and dose-toxicity study of SRT for BM [8], in our study, the α/β value was also set to 10. Due to the high heterogeneity of the PTV dose in SRT, to facilitate the dose effect analysis, we defined the nominal dose, peripheral dose, central dose and average dose as previously described [10]. Conducting dose-effect analyses of multiple BED parameters enables us to delve deeper into the factors that have a stronger correlation with tumor control.

### Probit analysis

Probit model regression analysis was conducted by XLSTAT 2016 (Addinsoft, Paris, France) as previously described [11, 12]. The statistical significance was set at the level of *P* value < 0.05. Subgroup analysis was conducted according to country, treatment era, proportion of male patients, median age, median tumour diameter, PTV margin, fractions and treatment technology. In order to analyze the dose-response relationship of tumor size subgroups, we conducted a spherical diameter conversion for the included studies that reported tumor volumes, as most BM exhibit spherical features.

## Results

### Description of the included studies

After a comprehensive search, no article about regression analysis on the dose effect relationship between dose and tumour local control rate based on published data was found. A total of 2377 potential related studies were identified using a systematic literature retrieval strategy. After removal of duplicates, 19 eligible studies involving 1523 patients in total and including six bibliographic references from relevant reviews, were obtained through title, abstract and full text screening and included in probit model regression analysis, as shown in Additional File 2: Fig. S1. These included studies were from 7 countries, with the most published studies coming from Japan (five), followed by Germany (four), the United States, Italy and France (three each), and the Netherlands (one). The main characteristics of the included studies are presented in Table 1.

**Table 1:** The main characteristics of the included studies

First Authors (Publication Year)	Country	Treatment Time	N. Patients (Proportion of Male)	N. Metastases	Median or mean Age	Most Common Histology	Tumour Size	Extracranial Disease Control (%)	GTV to PTV margin (mm)	Nominal/Peripheral/Central Dose (Gy)	Median No. of Fractions	Nominal/Peripheral/Central BED_10_ (Gy)	Treatment technology	Follow-up (months)	LC at 6 months (%)	LC at 12 months (%)
Pirzkall (1998) # [13]	Germany	1984–1997	158 (72%)	NR	57.4	NSCLC, Renal, Melanoma	20 (3–38) mm	NR	1–2	20/20/25	1	60/60/87.5	15MV, 9 NCA with CSC or manually driven MLC	NR	NR	89
Matsuo (1999) # [14]	Japan	1993–1998	51 (50%) 41	NR	60.2	NSCLC, SCLC, Breast, Colon, Liver, Sarcoma, Melanoma	NR	42	NR	20/20/25 25/25/50	1	60/60/87.5 87.5/87.5/300	10MV, 6–29 mm CSC in diameter	11 (1–44)	95 100	85 100
Aoyama (2003) [15]	Japan	1995–2000	87 (62%)	159	65	Lung cancer, Colon cancer, Breast cancer	3.3 (0.006–48.3) cc	45	2	35/32/35	4	65.6/57.6/65.6	6 MV or 10 MV, thermos-shell	6.3 (0.2–60.3)	85	81
Noel (2003) # [16]	France	1994–2002	34 (56%)	51	59	Adenocarcinoma, Squamous cell carcinoma	2.0 (0.1–16.5) cc	68	1	14/14/20.2	1	33.6/33.6/61.0	Leksell stereotactic head frame, 10MV,	29 (18–36)	90	78
Nakayama (2004) [17]	Japan	1999–2002	15 (73%)	20	69.5	NSCLC	NR	NR	2–3	40/40/44.4	4	80/80/93.7	10MV, 5 to 35 mm CSC	21 (2–34)	100	95
Narayana (2007) # [18]	U. S.	2004–2005	20 (45%)	20	60	Lung, Melanoma, Renal, Breast, Gastrointestinal	35 (20–50) mm	NR	3	30/30/-	5	48/48/-	6MV	10 (1–18)	90	70
Lutterbach (2008) # [19]	Germany	1994–2001	101 (49%)	155	59	Lung, Breast, kidney, Melanoma, Gastrointestinal	21 (4–30) mm	66.3	2	18/18/22.5	1	50.4/50.4/73.1	6MV, 6 NCA, CSC	NR	93	91
Fokas (2010) [20]	Germany	1996–2006	51 (70%)	NR	NR	Renal Cell Cancer	NR	NR	2–5	19/19/23.8	1	55.1/55.1/80.2	6MV, 7–11 noncoplanar static beams	9–95	NR	81
Nath (2010) [21]	U. S.	2005–2008	20 (38%)	NR	53	Breast, lung, Melanoma	NR	NR	1	18/18/20	1	50.4/50.4/60	6MV, 1000MU/min, dynamic MLC, 9–11 beams of fixed gantry	3 (0.2–21.3)	96	83
Saitoh (2010) [22]	Japan	2003–2006	49 (69%)	78	NR	Lung	NR	36.7	3	42/37.8/42	3	100.8/85.4/100.8	HeadFix, 6MV, 6–12 NCSB	17.4 (0.4–60.5)	NR	86
Fokas (2012) [23]	Germany	2000–2009	138 (43%)	NR	NR	NSCLC, Urogenital, Gastrointestinal	1.87 (0.03–11.17) cc	NR	2	20.0/20.0/-	1	60/60/-	Head mask, 6MV, 11–14 NCSB	28 (2.1–77)	84	73
Feuvret (2014) [24]	France	2007–2009	12 (50%)	12	57	Lung cancer, Breast cancer, Miscellaneous	32.61 (19.1-65.56) cc	67	2	33.0/23.1/33.0	3	69.3/40.9/69.3	6MV, 7 or 14 fixed conformal beams or 5 DCA	17.5 (2-61.3)	100	100
Yang (2014) [25]	U. S.	2000–2012	136 (NR)	186	58	Breast	9 (2–34) mm	46.3	2	21/21/26.3	1	65.1/65.1/95.2	Brown-roberts-Wells frame, 8–12 NCSB, 3-mm leaves MLC	23.4 (2.3-140.2)	95	90
Minniti (2016) # [26]	Italy	2008–2014	151 (49%) 138 (50%)	179 164	64 62	NSCLC, Breast cancer, Colon carcinoma, Melanoma	12.2 (4.4–32) cc 17.9 (5.6–54) cc	25 28	1–2	18.0/18.0/21.2 27.0/27.0/31.8	1 3	50.4/50.4/66.1 51.3/51.3/65.5	6–15 NCA or fixed beams	10	94 97	77 90
Navarria (2016) [27]	Italy	2011–2015	51 (62%) 51	51 51	61	NSCLC, Breast cancer, Melanoma	33.7 (9.2-122.3) cc	NR	3	27/27/27 32/32/32	3 4	51.3/51.3/51.3 57.6/57.6/57.6	VMAT	14 (3–53)	100 100	100 91
Aoki (2021) [28]	Japan	2016–2018	13 (77%)	113	67	Lung	2.0 ± 2.7 cc	NR	1	24/24/ -	1	81.6/81.6/-	6MV, 1000 MU/min, Arcs	17 ± 8.8	88	88
Badloe (2021) [29]	The Netherlands	2015–2016 2010–2012	37 (38%) 84 (51%)	NR	64	Lung, Breast, Melanoma, Renal cell, Colorectal	4.7 cc 10.9 cc	NR	0 2	21/21/27.6 18/18/23.6	1 1	65.1/65.1/103.8 50.4/50.4/79.3	6MV, NCA	31.2 (27.6–34.8) 79.2 (72-91.2)	97 89	84 87
Vigneau (2022) [30]	France	2016–2018	44 (57%)	61	64	NR	18.5 (4–56) mm	NR	1	23.1/23.1/33	3	40.9/40.9/69.3	THM, coplanar VMAT	31.9	93.2	90
Piras (2022) [31]	Italy	2016–2020	41 (46%)	57	68	NSCLC, Breast, Colorectal, SCLC, Melanoma	1.8 (0.1–22.4) cc	NR	3	30/30/31.6	5	48/48/51.3	THM	6 (1–21)	54.4	NR

# Study screened from review

N., Number; GTV, gross target volume; PTV, planning target volume; BED, biological equivalent dose; LC, local control; NR, not reported; NSCLC, non-small cell lung cancer; MV, megavoltage; NCA, non-coplanar arcs; CSC, circular shaped collimator; MLC, multi leaves collimator; DCA, dynamic conformal arc; NCSB, noncoplanar static beams; VMAT, volumatic modulated arc therapy; THM, thermoplastic head mask

### Probit analyses

The median of prescription dose was 23.1 Gy (range: 14–42 Gy), the median of fraction was one (range: 1–5), and the median of actuarial or rough 6-month and 12-month local control rates was 94% (range: 84–100%) and 86.5% (range: 70–100%), respectively.

**Fig. 1:**
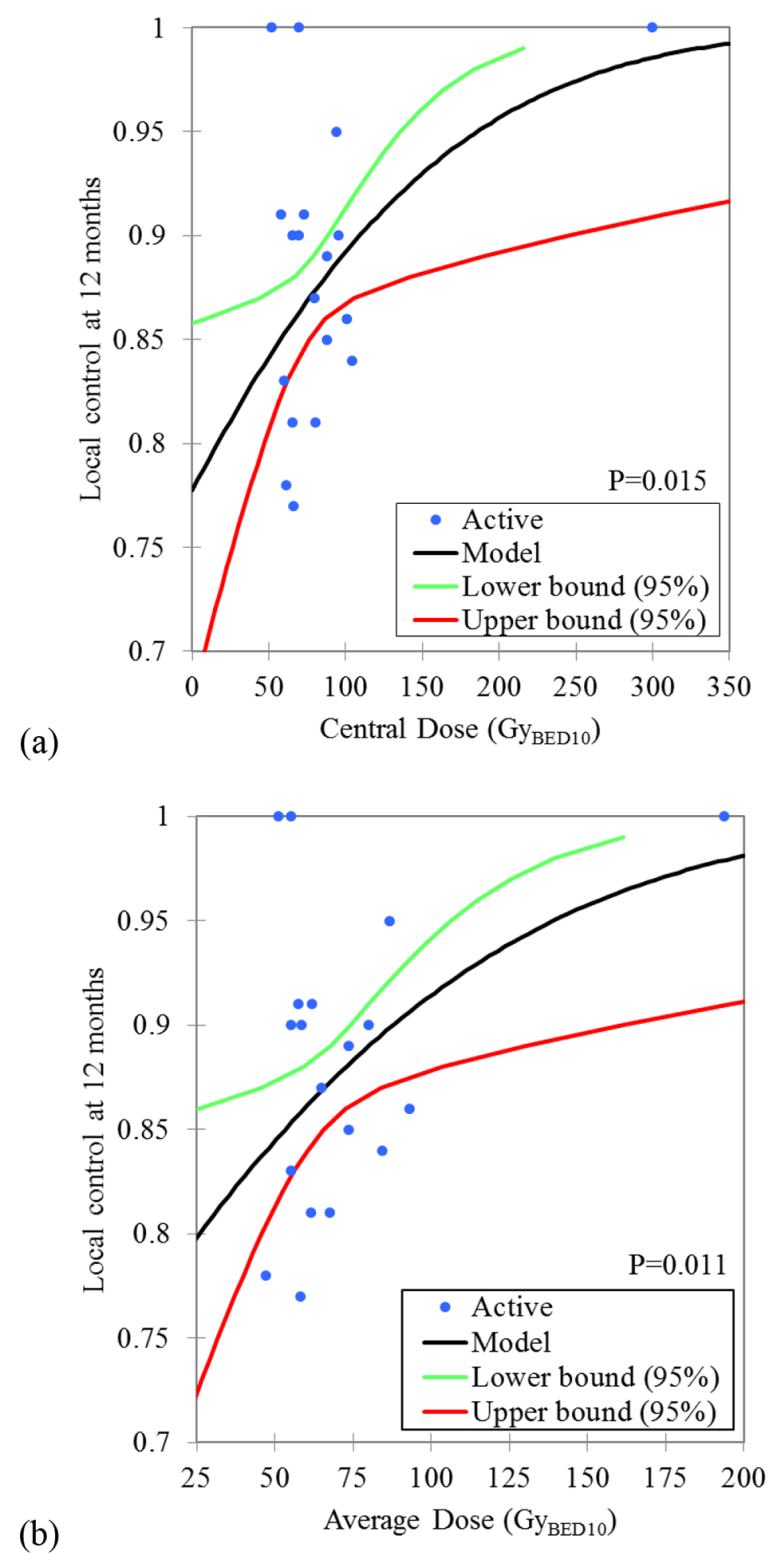
Dose-effect relationship between central **(a)** and average **(b)** biological equivalence dose (α/β = 10) and 12 months local control

For 12-month local control, there was no significant dose effect relationship between nominal BED_10_ and peripheral BED_10_ versus local control probability, with *P* values of 0.108 and 0.081, respectively. There were significant dose effect relationships between the centre BED_10_ and the average BED_10_ versus the 12-month local control probability, with *P* values of 0.015 and 0.011, respectively, as shown in Fig. 1. According to the model, the central BED_10_ and the average BED_10_ corresponding to probabilities of 90% 12-month local control were 109.2 Gy_BED10_ (95% confidence interval (CI): 88.7–245.9 Gy_BED10_) and 87.8 Gy_BED10_ (95% CI: 74.3–161.5 Gy_BED10_), respectively. A 12-month local control rate of 86.9% (95% CI: 81.7–89.7%) and 85.5% (95% CI: 81.2–89.2%) can be expected at centre BED_10_ of 80 Gy and an average BED_10_ of 60 Gy, respectively.

**Fig. 2:**
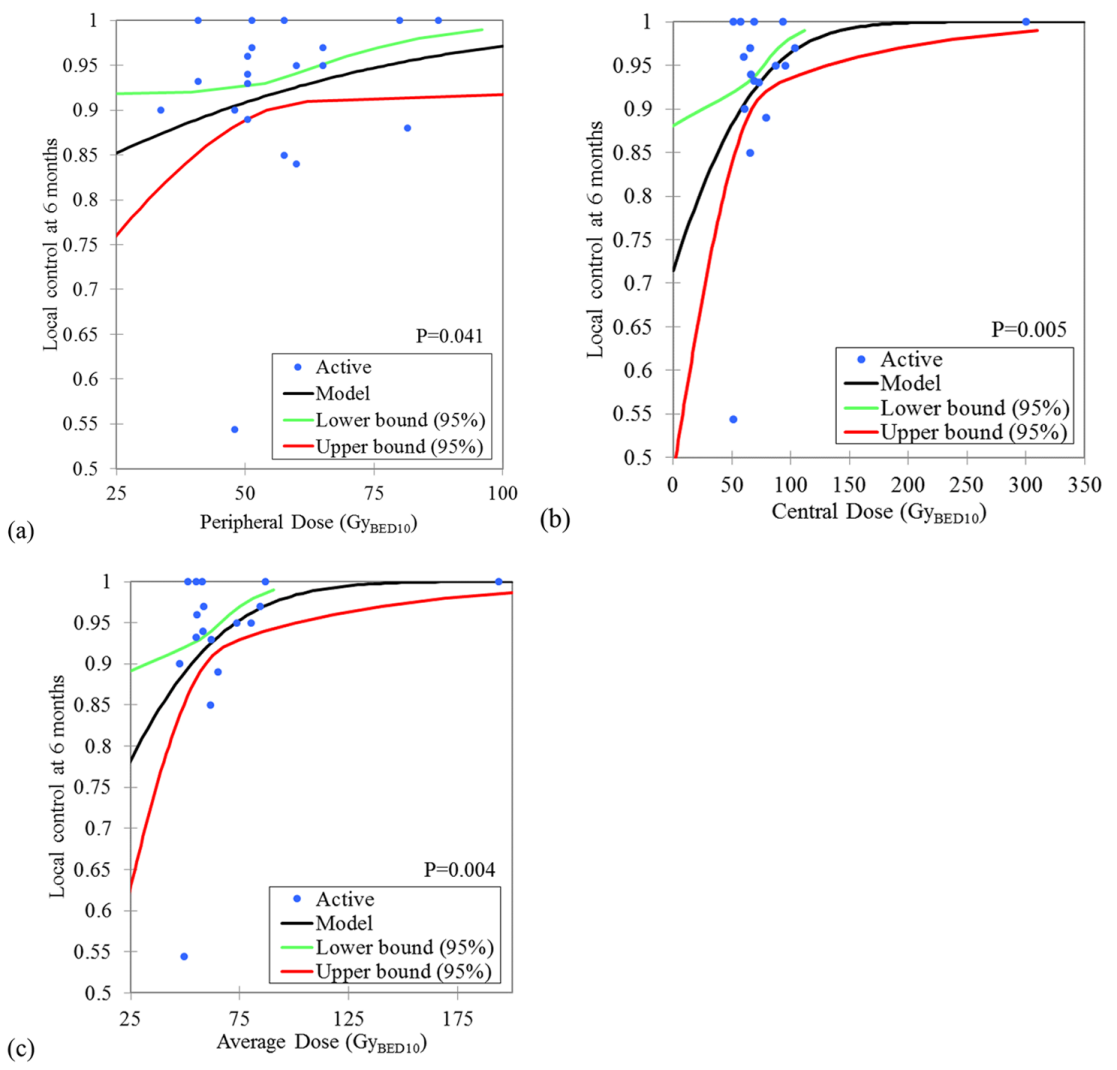
Dose-effect relationship between peripheral **(a)**, central **(b)** and average **(c)** biological equivalence dose ( α/β= 10) and 6 months local control

For 6-month local control, there was no significant dose effect relationship between nominal BED_10_ versus local control probability, with a *P* value of 0.074. There were significant dose effect relationships between the peripheral BED_10_, the centre BED_10_ and the average BED_10_ versus the 6-month local control probability, with *P* values of 0.041, 0.005 and 0.004, respectively, as shown in Fig. 2. According to the model, 6-month local control rates of 88.6% (95% CI: 83.8–92.2%), 93.8% (95% CI: 91.0–95.3%) and 91.8% (95% CI: 90.1–91.1%) can be expected at a peripheral BED_10_ of 40 Gy, a central BED_10_ of 80 Gy and an average BED_10_ of 60 Gy, respectively.

The Probit analyses based on subgroups show that the central BED_10_ has most cumulative number of significance (seven for 6-month and six for 12-month), followed by the average BED_10_ (seven for 6-month and five for 12-month), the nominal BED_10_ (five for 6-month and two for 12-month), and peripheral BED_10_ (three each for both 6-month and 12-month), as shown in Tables 2 and 3.

**Table 2:** The probit analyses based on subgroups between biological equivalence dose and 6-month local control

Parameter	Nominal BED_10_ (Gy_BED10_)			Peripheral BED_10_ (Gy_BED10_)			Central BED_10_ (Gy_BED10_)			Average BED_10_ (Gy_BED10_)		
	s (p)	ED90 (95% CI)	*P*	s (p)	ED90 (95% CI)	*P*	s (p)	ED90 (95% CI)	*P*	s (p)	ED90 (95% CI)	*P*
Country												
Japan	5 (212)	64.8 (-, -)	0.051	5 (212)	60.7 (46.9, 71.0)	**0.015**	4 (199)	73.0 (61.8, 90.6)	**0.016**	4 (199)	66.1 (59.5, 79.0)	**0.019**
Italy	5 (432)	50.0 (49.6, 50.4)	**< 0.0001**	5 (432)	50.0 (49.6, 50.4)	**< 0.0001**	5 (432)	57.2 (54.4, 60.6)	**< 0.0001**	5 (432)	54.3 (52.6, 55.7)	**< 0.0001**
Treatment era												
Before 2010	10 (524)	56.9 (-, -)	0.217	10 (524)	55.0 (-, -)	0.192	8 (366)	66.3 (-, -)	0.053	8 (366)	55.6 (-, -)	0.126
After 2005	11 (642)	45.2 (-80.3, 50.8)	**0.040**	11 (642)	42.7 (-, -)	0.121	10 (629)	53.6 (-415.8, 64.2)	**0.045**	10 (629)	52.9 (33.6, 57.3)	**0.013**
Proportion of male patients												
≤ 50%	11 (750)	52.0 (32.5, 59.5)	**0.023**	11 (750)	50.9 (-, -)	0.063	9 (592)	63.8 (58.2, 68.3)	**< 0.0001**	9 (592)	56.8 (52.5, 59.9)	**0.000**
> 50%	8 (379)	85.8 (-, -)	0.596	8 (379)	-2.4 (-, -)	0.796	7 (366)	76.9 (-, -)	0.099	7 (366)	75.2 (-, -)	0.470
Median age												
≥ 62	10 (517)	32.7 (-589.1, 46.1)	**0.042**	10 (517)	28.0 (-, -)	0.080	9 (497)	-79.0 (-, -)	0.284	9 (497)	-15.2 (-, -)	0.236
< 62	9 (615)	51.3 (-, -)	0.488	9 (615)	51.6 (-, -)	0.160	8 (602)	67.2 (62.6, 72.2)	**< 0.0001**	8 (602)	59.3 (55.7, 63.3)	**0.001**
Median tumour diameter *												
< 20 mm	8 (530)	73.6 (-, -)	0.072	8 (530)	69.8 (58.6, 452.5)	**0.042**	6 (379)	87.9 (80.1, 104.1)	**< 0.0001**	6 (379)	66.7 (61.3, 76.3)	**< 0.0001**
≥ 20 mm	8 (608)	49.3 (46.0, 50.1)	**0.007**	8 (608)	43.9 (-, -)	0.122	8 (588)	77.1 (73.2, 86.2)	0.001**	8 (588)	63.9, (61.9, 68.6)	0.001**
PTV margin												
≤ 2 mm	13 (995)	71.2 (-, -)	0.242	13 (995)	70.9 (-, -)	0.331	11 (844)	34.5 (-, -)	0.366	11 (844)	26.4 (-, -)	0.455
> 2 mm	5 (178)	48.2 (-, -)	0.993	5 (178)	48.2 (-, -)	0.993	4 (158)	51.6 (-, -)	0.993	4 (158)	49.8 (-, -)	0.992
Fractions												
1	11 (806)	41.1 (-, -)	0.195	11 (806)	41.1 (-, -)	0.195	9 (655)	47.4 (-, -)	0.110	9 (655)	45.9 (-, -)	0.065
2–5	9 (459)	47.2 (-, -)	0.347	9 (459)	48.2 (-, -)	0.151	8 (439)	59.3 (54.0, 64.9)	**0.001**	8 (439)	55.3 (52.3, 58.5)	**0.001**
Treatment technology												
CSC	4 (208)	47.0 (-, -)	0.102	4 (208)	47.0 (-, -)	0.102	4 (208)	65.5 (-, -)	0.334	4 (208)	55.9 (-, -)	0.269
MLC	8 (603)	-11.7 (-, -)	0.616	8 (603)	-240.8 (-, -)	0.910	8 (603)	125.7 (-, -)	0.176	8 (603)	117.8 (-, -)	0.355
Noncoplanar	7 (785)	77.9 (-, -)	0.523	7 (785)	77.9 (-, -)	0.523	6 (647)	-3145.8 (-, -)	0.989	6 (647)	-38.9 (-, -)	0.756
CNS			5			3			7			7

BED_10_, biological equivalence dose at α/β = 10; s (p), subgroup (patients); PTV, planning target volume; CSC, circular shaped collimator; MLC, multi leaves collimator; CNS, cumulative number of significances

Bold indicates that *P* < 0.05

*Brain metastasis volume converted to tumour diameter based on the sphere model

**Invalid dose effect relationship

**Table 3:** The probit analyses based on subgroups between biological equivalence dose and 12-month local control

Parameter	Nominal BED_10_ (Gy_BED10_)			Peripheral BED_10_ (Gy_BED10_)			Central BED_10_ (Gy_BED10_)			Average BED_10_ (Gy_BED10_)		
	s (p)	ED90 (95% CI)	*P*	s (p)	ED90 (95% CI)	*P*	s (p)	ED90 (95% CI)	*P*	s (p)	ED90 (95% CI)	*P*
Country												
Japan	6 (256)	89.2 (-, -)	0.127	6 (256)	76.4 (66.4, 138.6)	**0.020**	5 (243)	107.1(86.4,668.3)	**0.040**	5 (243)	91.6 (77.0, 183.5)	**0.016**
Italy	4 (391)	53.7 (-, -)	0.089	4 (391)	53.7 (-, -)	0.089	4 (391)	62.23(56.1,64.1)	0.003*	4 (391)	57.4 (-, -)	0.066
Treatment era												
Before 2010	13 (777)	96.7 (-, -)	0.054	13 (777)	90.3 (-, -)	0.054	11 (619)	99.0 (84.1, 265.7)	**0.027**	11 (619)	85.1 (72.6, 156.7)	**0.013**
After 2005	11 (737)	71.4 (-, -)	0.289	11 (737)	94.0 (-, -)	0.643	10 (724)	-28.3 (-, -)	0.726	10 (724)	-218.5 (-, -)	0.931
Proportion of male patients												
≤ 50%	10 (709)	80.0 (-, -)	0.062	10 (709)	80.0 (-, -)	0.062	8 (551)	102.9 (82.3, 947.3)	**0.042**	8 (551)	83.1 (69. 3, 340.9)	**0.034**
> 50%	11 (564)	-704.1 (-, -)	0.966	11 (564)	108.2 (-, -)	0.618	10 (629)	25.1 (-, -)	0.555	10 (629)	-168.6 (-, -)	0.914
Median age												
≥ 62	8 (569)	-1220.7 (-, -)	0.974	8 (569)	171.4 (-, -)	0.764	7 (556)	125.6 (-, -)	0.425	7 (556)	117.1 (-, -)	0.551
< 62	11 (675)	57.8 (49.8, 70.2)	**0.004**	11 (675)	57.7 (46.6, 79.1)	**0.017**	10 (655)	80.0 (-, -)	0.089	10 (655)	68.1 (-, -)	0.072
Median tumour diameter **												
< 20 mm	7 (610)	157.1 (-, -)	0.621	7 (610)	120.1 (-, -)	0.447	5 (459)	92.7 (81.0, 201.8)	**0.026**	5 (459)	80.4 (69.1, 5208.3)	**0.049**
≥ 20 mm	9 (645)	60.0 (-, -)	0.214	9 (645)	174.9 (-, -)	0.929	8 (625)	50.2 (-, -)	0.159	8 (625)	47.1 (-, -)	0.209
PTV margin												
≤ 2 mm	12 (1153)	211.9 (-, -)	0.749	12 (1153)	781.7 (-, -)	0.948	10 (1002)	98.2 (83.7, 951.9)	**0.044**	10 (1002)	84.9 (-, -)	0.078
> 2 mm	6 (237)	-29.5 (-, -)	0.850	6 (237)	-619.9 (-, -)	0.985	5 (217)	77.4 (-6.7,104.2)	0.034*	5 (217)	70.7 (-, -)	0.089
Fractions												
1	13 (1015)	76.4 (66.6, 125.7)	**0.005**	13 (1015)	76.4 (66.6, 125.7)	**0.005**	11 (864)	98.5 (87.8, 141.0)	**0.003**	11 (864)	82.0 (73.8, 109.0)	**0.001**
2–5	9 (467)	40.9 (-, -)	0.398	9 (467)	39.3 (-, -)	0.475	8 (447)	65.2 (-, -)	0.199	8 (447)	59.6 (-, -)	0.199
Treatment technology												
CSC	5 (366)	58.1 (-, -)	0.075	5 (366)	58.1 (-, -)	0.075	5 (366)	87.7 (-, -)	0.124	5 (366)	73.2 (-, -)	0.085
MLC	9 (761)	63.4 (-, -)	0.119	9 (761)	69.6 (-, -)	0.374	9 (761)	210.6 (-, -)	0.841	9 (761)	106.9 (-, -)	0.655
Noncoplanar	9 (885)	412.5 (-, -)	0.843	9 (885)	559.0 (-, -)	0.914	8 (747)	122.5 (-, -)	0.281	8 (747)	108.5 (-, -)	0.344
CNS			2			3			6			5

BED_10_, biological equivalence dose at α/β = 10; s (p), subgroup (patients); PTV, planning target volume; CSC, circular shaped collimator; MLC, multi leaves collimator; CNS, cumulative number of significances

Bold indicates that *P* < 0.05

*Invalid dose effect relationship

**Brain metastasis volume converted to tumour diameter based on the sphere model

## Discussion

The probability of cancer patients developing BM diseases is high, and there is a growing trend due to the support of systematic treatment and high-resolution imaging. SRT is an attractive alternative treatment option that may avoid these side effects and improve local tumour control. With the popularization of various high technologies, the application of LINAC-based SRT for BM is becoming increasingly widespread. However, there is no consensus on the prescription dose and dose heterogeneity requirements within the target volume of LINAC-based SRT for patients with BM. It is meaningful that our study tried to find the optimal prescription dose and dose heterogeneity of LINAC-based SRT for BM using probit model regression analysis based on published data.

In this study, there was no significant dose-response relationship between the nominal BED_10_ and the 6-month or 12-month local control, mainly due to the lack of universal representativeness of the nominal dose. There was no uniform standard for the prescription dose method of SRT for patients with BM. Some prescription doses were defined at the minimum dose [14, 16, 18, 29, 31], and some were defined at the isocentre [15, 24]. At the same time, the degree of dose heterogeneity in PTV for different studies may be diverse, even if the same clinical trial protocol is followed. For example, in the RTOG 90 − 05 protocol, the prescribed dose was defined as an isodose line of 50–90%, which meant that the maximum dose was 111–200%, with significant differences. One study among the included studies, set the prescribed dose at a 50% isodose line [14], while some set it at 80% [13, 14, 19, 20, 25], and others at 90% [17, 21] and even 95% [31]. Therefore, for the same nominal dose, it may occur that the actual dose given to PTV is not comparable.

To address this issue, we selected peripheral BED_10_, central BED_10_, and average BED_10_ to establish the dose-response relationship in this study. Interestingly, in this study, there were significant dose-effect relationships between the centre BED_10_ and the average BED_10_ and the 12-month local control probability. For 6-month local control, there were significant dose-effect relationships between the peripheral BED_10_, the centre BED_10_ and the average BED_10_ and the 6-month local control probability. Although there has been no report on dose-response relationships for patients with BM treated with LINAC-based SRT, some scholars have explored the dose-response relationship, including those based on LINAC, Gamma knife, and Cyberknife [6, 8, 9, 32], as well as confusion with other tumours [7]. These dose-effect relationships are to some extent comparable to our results.

In our study, the peripheral BED_10_ corresponding to probabilities of 90% 6-month local control were 45.5 Gy_BED10_. Amsbaugh et al. [32] used a proportional hazards modelling to determine the dose-volume response relationship between the ratio of maximum lesion dose per mm-diameter (Gy/mm) versus local control for frameless SRT based on 316 BMs from 121 patients. This study followed the RTOG 90 − 05 protocol, and the prescription doses, which is the peripheral dose defined in our study, were used in the quantitative dose effect analysis. The results showed that local control of 80%, 85%, and 90% corresponded to maximum doses per millimetre of 1.67 Gy/mm, 2.86 Gy/mm, and 4.4 Gy/mm, respectively. In order to make the results comparable, we conducted diameter conversion based on the sphere model for the included studies that reported tumour volumes. The median calculated tumour diameter for 12 included studies reporting the tumour volume was 25.8 mm, the other four reported tumour diameters with a median of 17 mm; therefore, the overall average was 23.6 mm. Considering the diameter of the tumour, 6-month local control of 90% corresponded to BED_10_ per millimetre of 1.93 Gy_BED10_/mm.

Redmond et al. [6] established a tumour control probability model after SRT for BMs by screening for published articles on dosimetric and tumour control data. The model results showed that for tumours ≤ 20 mm, single-fraction doses of 18 and 24 Gy corresponded with > 85% and 95% 1-year LC rates, respectively. For tumours 21 to 30 mm, an 18 Gy single-fraction dose was associated with 75% LC. For tumours 31 to 40 mm, a 15 Gy single-fraction dose yielded ∼69% LC. For 3- to 5-fraction FSRT using doses in the range of 27 to 35 Gy, 80% 1-year LC has been achieved for tumours of 21 to 40 mm in diameter. The results of this study are based on Gamma Knife, Cyberknife, and LINAC-based SRT. This result indicated that tumour diameter plays an important role in tumour control in SRT for BMs.

Loo et al. [33] discussed the dose-effect relation in BMs treated by SRT accounting for fractionation and technical considerations. A BED_10_ of 40 to 50 Gy seemed associated with a 12-month local control rate > 70%. A BED_10_ of 50 to 60 Gy seemed to achieve a 12-month local control rate at least of 80% at 12 months. In our study, 12-month local control rates of 86.9% and 85.5% could be expected at a centre BED_10_ of 80 Gy and an average BED_10_ of 60 Gy, respectively. Based on the definitions of central BED_10_ and average BED_10_, it can be roughly concluded that with the assurance of higher doses given to the PTV centre, 85% 12-month local control can be expected at a peripheral BED_10_ of approximately 40 Gy. This dose seemed to be lower than the results of Loo et al., but our study emphasized the higher dose to the centre and average PTV.

Shuryak et al. [7] analysed published tumour control data for lung tumours and BMs and obtained dose-response relationships based on several radiobiological models. Fortunately, this study provided a dose-response relationship for the BM subgroup. It is worth noting that the isocentre dose was used for quantitation in the dose effect analysis. Although the dose-response relationship of the BM subgroup did not use a radiobiological model, BED conversion based on a single fraction curve can be compared with the results of this study. In our study a 12-month local control rate of 86.9% could be expected at the centre BED_10_ of 80 Gy, which is equivalent to 23.7 Gy for single fraction schedule. In Shuryak’s study, 87.5% local control probability can be expected at 23.7 Gy for single fraction dose-effect curve. Therefore, the model results of these two studies are consistent.

Wiggenraad et al.‘s dose-response results by eye fitting based on published literature showed that a BED_12_ of at least 40 Gy was associated with a 12–month local control rate of 70% or more [9]. This study used the LQC model for biological dose conversion and a α/β value of 12. The BED based on the LQC model is slightly smaller than that based on the LQ model, and BED12 is slightly smaller than BED10. The degree of reduction in both depends on the dose per fraction. These limitations limit the comparability of the study.

This study has some limitations that have been discussed in previous articles [10, 11]. Besides, BMs belong to the advanced stage of patients with cancer, and tumour control is influenced by many factors, such as primary tumour control, extracranial metastases, and pathology. Furthermore, the selection of α/β value is also a limitation of this study. The α/β value has been a core parameter in calculating BED in the LQ model, and there has always been controversy. Brain metastasis originates from different tumor sites and pathological types, exhibiting different α/β values, leading to a more complex scenario. For instance, melanoma, sarcoma, breast cancer, and prostate cancer are known to have lower α/β values compared to other tumors. It is worth noting that the proportion of BM originating from these specific primary tumors in the included studies in this analysis is relatively small (347/1523, 22.8%). Several studies on BM uses an α/β value of 10 [8, 34], even higher [9, 35], to calculate the BED. Consequently, while the issue of α/β value selection is a limitation, its impact on the overall conclusion of the study is somewhat limited.

In conclusion, for patients with BM treated with LINAC-based SRT, more attention should be given to the central dose and average dose of PTV. A 12-month local control rate of 86.9% (95% CI: 81.7–89.7%) and 85.5% (95% CI: 81.2–89.2%) can be expected at a centre BED_10_ of 80 Gy and an average BED_10_ of 60 Gy, respectively. A 6-month local control rate of 88.6% (95% CI: 83.8–92.2%), 93.8% (95% CI: 91.0–95.3%) and 91.8% (95% CI: 90.1–91.1%) can be expected at a peripheral BED_10_ of 40 Gy, a centre BED_10_ of 80 Gy and an average BED_10_ of 60 Gy, respectively. A clear definition of the dose prescription should be established to ensure the effectiveness and comparability of treatment.

### Electronic supplementary material

Below is the link to the electronic supplementary material.

Supplementary Material 1

Supplementary Material 2

## Data Availability

All data, models, or code generated or used during the study are available from the corresponding author by request.
