# Supplementary material for: Dose-effect relationship of linear accelerator based stereotactic radiotherapy for brain metastases
Source: Radiat Oncol. 2023 Oct 30;18:177. doi: 10.1186/s13014-023-02360-y (PMC10617179; doi:10.1186/s13014-023-02360-y)
Supplement: Supplementary file 1 — Supplementary Material 1 [file 13014_2023_2360_MOESM1_ESM.docx]

Table S1. Search strategies of databases

| Databases | Search strategies | Search outcomes |
| --- | --- | --- |
| The PubMed Database | #1 Search: stereotactic radiosurgery[Title]  #2 Search: stereotactic radiotherapy[Title]  #3 Search: hypofractionated radiosurgery[Title]  #4 Search: stereotactic body radiotherapy[Title]  #5 Search: stereotactic ablative radiotherapy[Title]  #6 Search: SRT[Title]  #7 Search: SRS[Title]  #8 Search: SBRT[Title]  #9 Search: SABR[Title]  #10 Search: #1 OR #2 OR #3 OR #4 OR #5 OR #6 OR #7 OR #8 OR #9  #11 Search: (brain metastases[Title]) OR (brain metastasis[Title])  #12 Search: #10 AND #11 Filter: Language: English | 3,479  1,537  12  1,966  485  187  808  1,154  290  9,441  9,023  1,047  1,024 |
| Web of Science Core Collection | #1 TI=(stereotactic radiosurgery)  #2 TI=(stereotactic radiotherapy)  #3 TI=(hypofractionated radiosurgery)  #4 TI=(stereotactic body radiotherapy)  #5 TI=(stereotactic ablative radiotherapy)  #6 TI=(SRT)  #7 TI=(SRS)  #8 TI=(SBRT)  #9 TI=(SABR)  #10 #1 OR #2 OR #3 OR #4 OR #5 OR #6 OR #7 OR #8 OR #9 #11 TI=(brain metastases) OR TI=(brain metastasis) #12 #10 AND #11 Filter: Language: English Filter: Document Types: Article or Review Article | 5,594  7,564  173  3,534  1,061  567  2,233  5,033  958  20,026  13,517  2,042  2,002  893 |
| The Cochrane Library | #1 (stereotactic radiosurgery): ti, ab, kw  #2 (stereotactic radiotherapy): ti, ab, kw  #3 (hypofractionated radiosurgery): ti, ab, kw  #4 (stereotactic body radiotherapy): ti, ab, kw  #5 (stereotactic ablative radiotherapy): ti, ab, kw  #6 (SRT): ti, ab, kw  #7 (SRS): ti, ab, kw  #8 (SBRT): ti, ab, kw  #9 (SABR): ti, ab, kw  #10 #1 OR #2 OR #3 OR #4 OR #5 OR #6 OR #7 OR #8 OR #9  #11 (brain metastases): ti, ab, kw OR (brain metastasis): ti, ab, kw  #12 #10 AND #11 | 802  1,561  48  887  289  798  1,164  900  259  3,605  2,927  460 |
