# Supplementary figures and images for: Dose-effect relationship of linear accelerator based stereotactic radiotherapy for brain metastases
Source: Radiat Oncol. 2023 Oct 30;18:177. doi: 10.1186/s13014-023-02360-y (PMC10617179; doi:10.1186/s13014-023-02360-y)

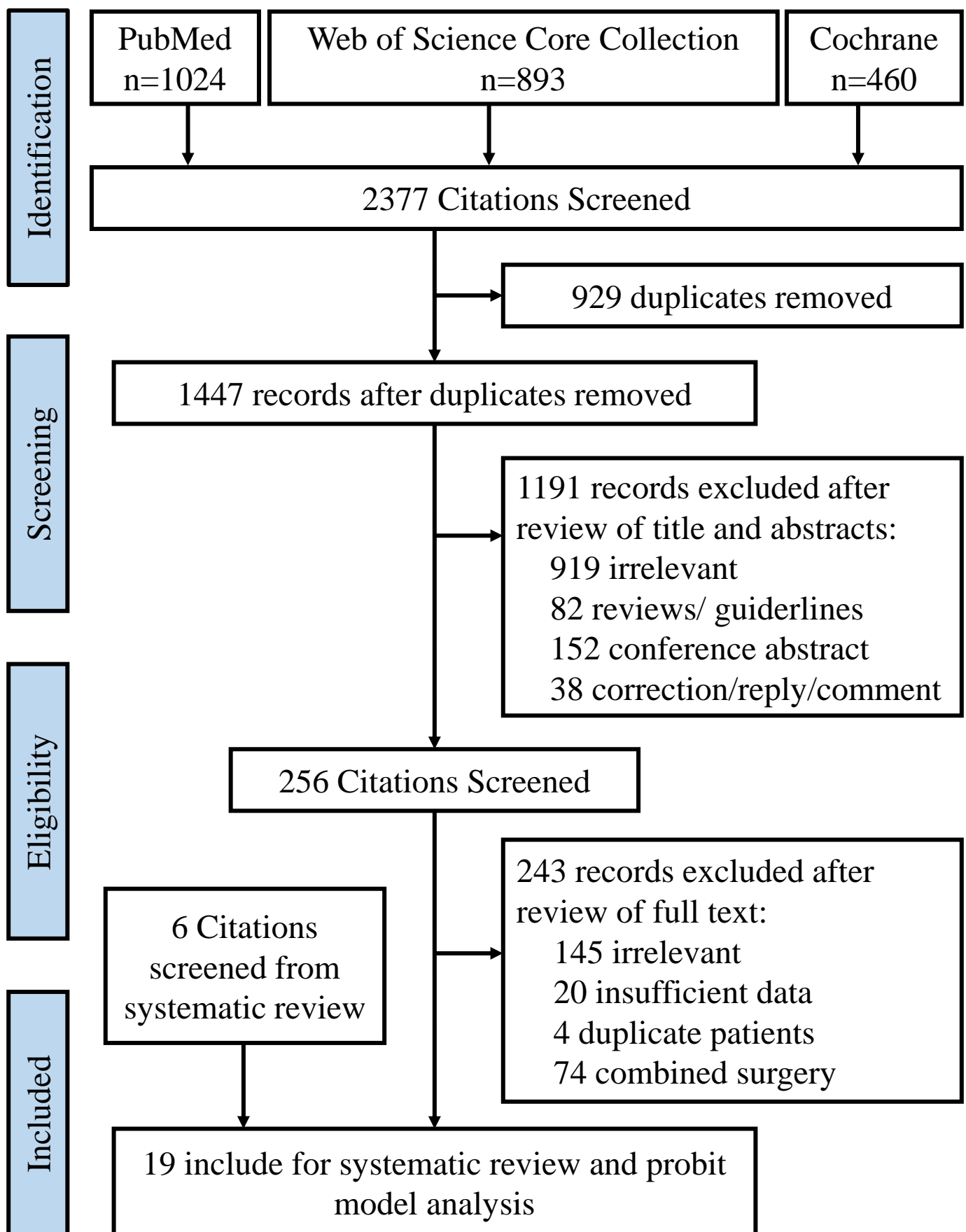

Figure S1. PRISMA Flow diagram of the included studies.

Supplement: Supplementary file 2 — Supplementary Material 2 [file 13014_2023_2360_MOESM2_ESM.pdf]
